# Dlf1, a WRKY Transcription Factor, Is Involved in the Control of Flowering Time and Plant Height in Rice

**DOI:** 10.1371/journal.pone.0102529

**Published:** 2014-07-18

**Authors:** Yuhui Cai, Xujun Chen, Ke Xie, Qikai Xing, Yawen Wu, Jing Li, Caihong Du, Zongxiu Sun, Zejian Guo

**Affiliations:** 1 Key Laboratory of Plant Pathology, China Agricultural University, Beijing, China; 2 China National Rice Research Institute, Hangzhou, China; 3 Zhejiang University, Hangzhou, China; Institute of Genetics and Developmental Biology, Chinese Academy of Sciences, China

## Abstract

Flowering time and plant height are important agronomic traits for crop production. In this study, we characterized a semi-dwarf and late flowering (*dlf1*) mutation of rice that has pleiotropic effects on these traits. The *dlf1* mutation was caused by a T-DNA insertion and the cloned *Dlf1* gene was found to encode a WRKY transcription factor (OsWRKY11). The *dlf1* mutant contains a T-DNA insertion at the promoter region, leading to enhanced accumulation of *Dlf1* transcripts, resulting in a semidominant mutation. The *dlf1* mutation suppressed the transcription of *Ehd2/RID1/OsId1* and its downstream flowering-time genes including *Hd1*, *Ehd1* and *Hd3a* under both long-day (LD) and short-day (SD) conditions. Knock-down of *Dlf1* expression exhibited early flowering at LD condition related to the wild-type plants. Accumulation of *Dlf1* mRNA was observed in most tissues, and two splicing forms of *Dlf1* cDNAs were obtained (*OsWRKY11.1* and *OsWRKY11.2*). These two proteins showed transactivation activity in yeast cells. Dlf1 protein was found to be localized in the nucleus. Enhanced expression of *OsWRKY11.2* or its 5′ truncated gene showed similar phenotypes to the *dlf1* mutant, suggesting that it might function as a negative regulator. We conclude that Dlf1 acts as a transactivator to downregulate *Ehd2/RID1/OsId1* in the signal transduction pathway of flowering and plays an important role in the regulation of plant height in rice.

## Introduction

Increasing cereal output has been a fundamental goal to meet the soaring demand for food. Plant height, potential yield and flowering time are important traits for cereal production. Plant height, one of the main selection trait in rice (*Oryza sativa*) breeding, is controlled mostly by genes related to the synthesis and regulation of phytohormones, such as gibberellin (GA) and brassinolide [1]–[3]. Potential yield per rice plant is determined by grain weight, and numbers of grains per panicle and tillers per plant [4]–[6]. Flowering time of plants is controlled by both environmental and developmental factors, with photoperiod as an important environmental signal. Molecular genetic analysis in *Arabidopsis thaliana*, a long-day (LD) plant, has identified a set of key regulators functioning in the photoperiod-mediated flowering pathway. For example, the nuclear protein CONSTANS (CO) positively regulates the flowering activator *FLOWERING LOCUS T* (*FT*), which further interacts with the bZIP transcription factor *FLOWERING LOCUS D* (*FD*) to control flowering time in *Arabidopsis* [7]–[9]. *SUPPRESSOR OF OVEREXPRESSION OF CONSTANS1* (*SOC1*), encoding a MADS box transcription factor, is activated by CO through FT and repressed by FLC (FLOWERING LOCUS C) via direct binding to the promoter [10].

Analysis of natural variants and mutants in rice, a short-day (SD) plant, has revealed the existence of a genetic pathway similar to that in *Arabidopsis* in photoperiodic flowering. *Heading date 1* (*Hd1*), *Heading date 3a* (*Hd3a*), *Heading date 6* (*Hd6*) and *OsGI* in rice are orthologs of *Arabidopsis CO*, *FT*, the α-subunit of kinase CK2, and *GIGANTEA* (*GI*), respectively. *OsGI*, a gene involved in the circadian clock control, regulates *Hd1* and *Hd3a* in photoperiodic flowering, which promotes flowering under SD conditions and suppresses it under LD conditions [11]–[14]. In addition, *early heading date 1* (*Ehd1*), a B-type response regulator that is specific to floral induction in rice, regulates the expression of *Hd3a*, *FTL1* and *OsMADS14* [15]. *Ehd1* functions upstream of *Hd3a* and *RFT1* through the *Hd1*-independent pathway. *Ehd2/RID1*/*OsId1* were isolated separately by three groups and the locus was found to encode a Cys2/His2-type zinc finger transcription factor orthologous to the *INDETERMINATE 1* (*ID1*) gene in maize [16]–[18]. Loss-of-function *ehd2/rid1/Osid1* mutants were never- or extremely late-flowering in comparison with wild-type plants under both SD and LD conditions. Functional analysis revealed that *Ehd2/RID1/OsId1* acts as a master switch and promoter of phase transition mainly by regulating *Ehd1* and the downstream genes. Further, a CCT (CO, CO-LIKE and TIMING OF CAB1)-domain protein encoding gene *Ghd7*, which was uncovered from a natural variant rice, suppresses the expression of *Ehd1* and *Hd3a* genes under LD conditions but does not affect *Hd3a* expression under SD conditions [19]. Recently, *Hd17/Ef7*, an ortholog of *Arabidopsis EARLY FLOWERING 3* (*ELF3*), has been characterized to promote rice flowering by repressing *Ghd7* expression under both LD and SD conditions [20], [21]. In addition, *Ehd3*, which encodes a protein containing two plant homeodomain (PHD) finger motifs, functions also as a LD suppressor of *Ghd7* [22]. The mutation in *DTH8/Ghd8/Hd5* shows pleiotropic effects on grain number, plant height and heading date, and causes delayed flowering by down-regulation of *Ehd1* under LD conditions [23]–[25]. On the other hand, *OsMADS51* is a SD promoter functioning downstream of *OsGI1* and upstream of *Ehd1* [9], [26], whereas *Ehd4*, encoding a CCCH-zinc finger regulator, promotes rice flowering by stimulating the expression of *Ehd1* and its downstream genes [27].

Much progress has been achieved in the genetic dissection of photoperiodic flowering of rice, but the molecular regulation is still largely unknown. In this study, we characterized a semi-dwarf and late flowering (*dlf1*) mutant and identified *Dlf1* gene that encodes a WRKY transcription factor. Our results showed that overexpression of *Dlf1* suppressed flowering by inhibiting the expression of *Ehd2/RID1/OsId1* under both LD and SD conditions and influenced plant height in rice.

## Results

### Isolation and morphological characterization of the *dlf1* mutant

**Figure 1:**
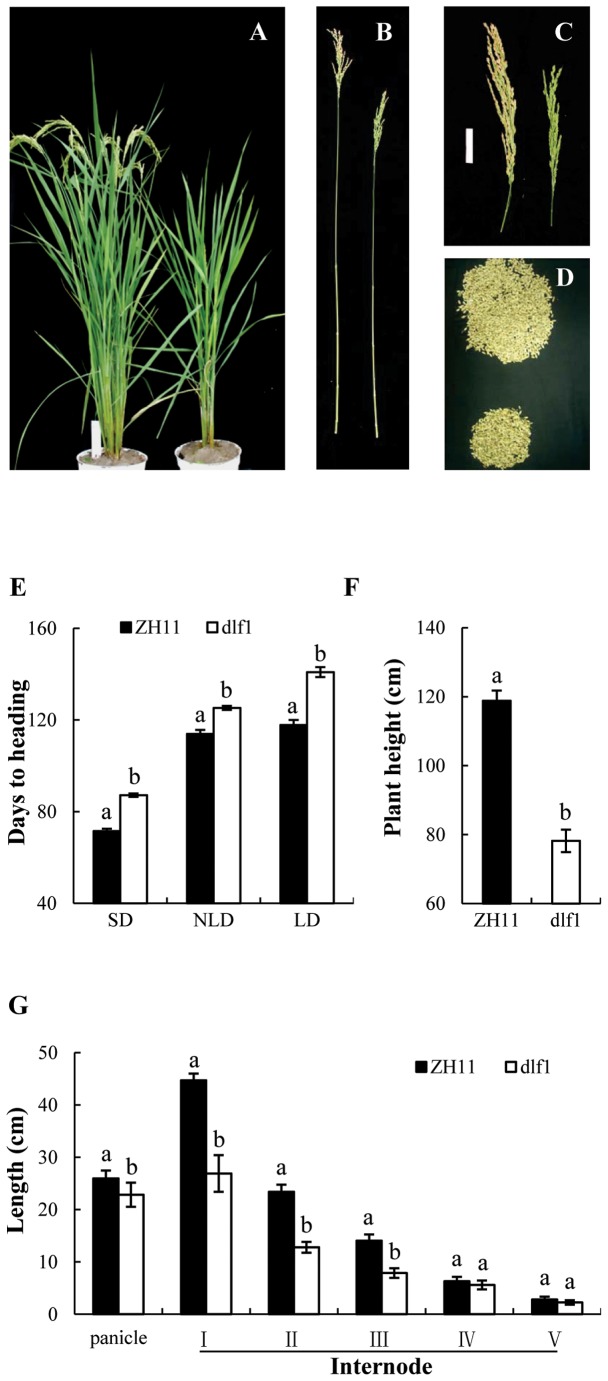
Phenotypes of Zhonghua 11 (ZH11) and *dlf1* mutant. (**A**) Photos of the wild-type ZH11 (left) and mutant *dlf1* (right), taken when ZH11 reached maturity. (**B**) Photos of main culms of ZH11 (left) and *dlf1* (right). (**C**) Main panicles of ZH11 (left) and *dlf1* (right). (**D**) Grains from main panicles of ZH11 (top) and *dlf1* (bottom). (**E**) Days to heading of ZH11 and *dlf1* under SD, LD and NLD (natural LD) conditions. Data are represented as mean values ± standard derivation (SD) of 20 plants. (F) Plant heights of ZH11 and *dlf1* under natural LD conditions. Values are means ± SD, n = 20. Experiment were performed three times, showing similar results. (**G**) Difference of internode lengths between matured ZH11 and *dlf1* plants. The plants were grown in the experimental field under natural LD conditions. Values are means ± SD, n = 20. The same experiments were repeated three times, and the similar results were obtained. For SD and LD treatments, the plants were grown in greenhouse under natural light conditions and shaded at the time designated. a and b in figure indicate ranking by Duncan test at P<0.05, starting from a. b is significantly different from a.

A semi-dwarf and late flowering (*dlf1*) mutant was identified from a T_1_ population of T-DNA insertion lines of cultivar *Oryza sativa* L. *japonica* Zhonghua 11 (ZH11). The flowering time of *dlf1* plants was delayed for about two weeks compared with wild-type plants in the experimental field in late May, 2003 in Hangzhou, China (Fig. 1A). DNA blot analysis showed that there was only one copy of T-DNA insertion in the mutant line (data not shown).

To determine whether the heading time of the *dlf1* mutant differed under different photoperiod conditions, the mutant and wild-type plants were grown under SD conditions (10/14 h light/dark) and LD conditions (14/10 h light/dark). Heading time of the *dlf1* mutant plants was 87.2±1.0 d, which was delayed ca. 16 d in comparison with wild-type (71.5±0.8 d) under SD conditions (Fig. 1E). Under LD conditions, heading time of the *dlf1* mutant plants was 140.9±2.2 d and increased by ca. 23 d compared with ZH11 (117.8±2.2 d). Other phenotypes differed significantly under natural LD conditions, including homozygous *dlf1* mutant lowered plant height (Figs. 1A and F) caused by reducing cell size (Fig. S1A), and decreased number of spikelets per panicle (Fig. S1B). The *dlf1* mutant plants also had less 1000-grain weight (Fig. S1C) and showed leaf rolling phenotypes (Figs. S1D and E) under natural LD conditions.

### *Dlf1* encodes a WRKY transcription factor

**Figure 2:**
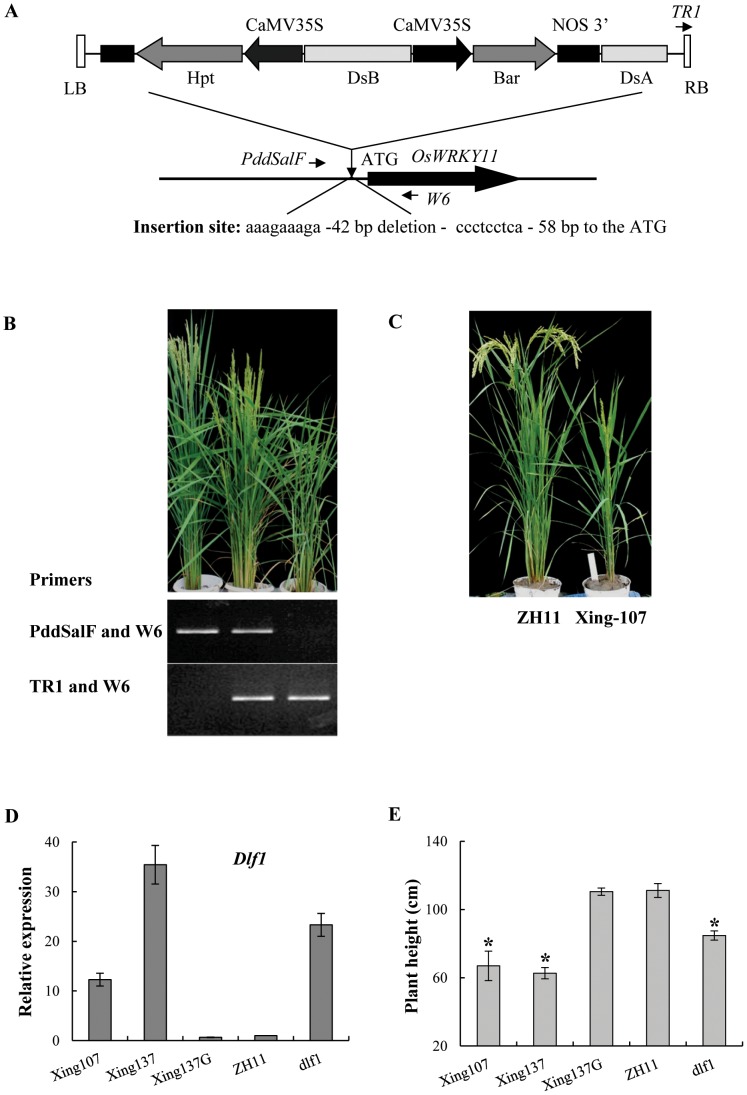
Molecular features of *Dlf1*. (**A**) Structure of T-DNA insertion site. The perpendicular arrow head indicates the insertion site. The T-DNA was inserted into the upstream of *OsWRKY11*. Arrows indicate the primers used for analyzing the insertion site. LB and RB represent the left and right borders of T-DNA. (**B**) PCR genotyping *Dlf1* segregants in F_2_ with primers as indicated in **A** (showed in *italics*). Primers PddSalF and W6 amplify a product from wild-type. PCR-positive plants with primers of TR1 and W6 indicate T-DNA insertion in the examined site and a homozygote for the insertion if without an amplification product from PddSalF and W6. (**C**) Photos of ZH11 (left) and *Cp-Ins-Dlf1* transgenic T_1_ plant (named as Xing-, right), taken when ZH11 reached maturity. (**D**) Expression of *Dlf1* in transgenic plants of T_1_ progenies under natural LD conditions. The first and second youngest leaves were sampled from 90-d-old plants for RNA isolation. Transcription levels were quantified by quantitative reverse-transcription PCR (qPCR) and the gene expression was normalized to rice *ubiquitin* gene (*Ubq*) for each sample. Transcription levels are expressed as ratio to the level of transcript in ZH11. (**E**) Plant heights. Xing-, the transgenic plants, the suffix G for segregated non-transgenic plants; ZH11, the control and *dlf1*, the mutant. The plants were grown in the experimental field under natural LD conditions. Values are shown as means ± SD of two independent experiments. Asterisks indicate significant difference between ZH11 and other lines (P<0.05, Duncan test).

Using the thermal asymmetric interlaced PCR (Tail-PCR) method, we isolated the genomic DNA flanking of the T-DNA insertion site from the *dlf1* mutant. A BLAST search of the flanking sequence revealed that the T-DNA was inserted at 67 bp upstream of the initial ATG (the ‘A’ was defined as +1) of the predicted coding sequence of the gene *OsWRKY11* (LOC_Os01g43650, AK108745; and named as *Dlf1* in the present study) (Fig. 2A). The flanking sequence of the other T-DNA border was amplified and revealed a 42 bp deletion at the insertion site.

To ascertain whether the *dlf1* phenotypes are associated with the T-DNA insertion, genetic analysis was performed using two filial populations of reciprocal crosses between the *dlf1* mutant and wild-type plants. The flowering time and the plant heights co-segregated in a manner of fit the 1∶2∶1 population (data not shown). Correspondingly, the genotypes of the T-DNA insertion in the same populations were determined by PCR and showed a tight linkage with the phenotypes (Fig. 2B). These results indicated that the *Dlf1* is semi-dominant and the T-DNA insertion event upstream of the *dlf1* gene is responsible for the mutant phenotype.

To examine the effect of the T-DNA insertion on the gene expression, the *Dlf1* total expression level was assayed by quantitative real-time PCR (qPCR). The transcript levels of *Dlf1* in rice leaves were enhanced significantly in the *dlf1* mutant compared with the wild-type ZH11 (Figs. 2D). To verify that the mutant phenotypes were caused by the high expression of *Dlf1* that was related to the T-DNA insertion, a fragment from the T-DNA insertion site to the end of the *Dlf1* coding region (*Cp-Ins-Dlf*) were transformed into the wild-type background. Among 14 plants regenerated (named as Xing-), 10 showed variation in dwarfism, and late flowering phenotypes in the T_0_ progeny (data not shown). Two lines were used for further analysis in the T_1_ generation. The levels of *Dlf1* expression were higher in dwarf plants compared with the wild-type and segregated non-transgenic plants (Figs. 2C, D and E). The dwarf plants also showed delayed flowering for about two weeks late under natural LD conditions, indicating that the increase of *Dlf1* expression caused the *dlf1* phenotypes.

### *Dlf1* is ubiquitously expressed and alternatively spliced in rice

**Figure 3:**
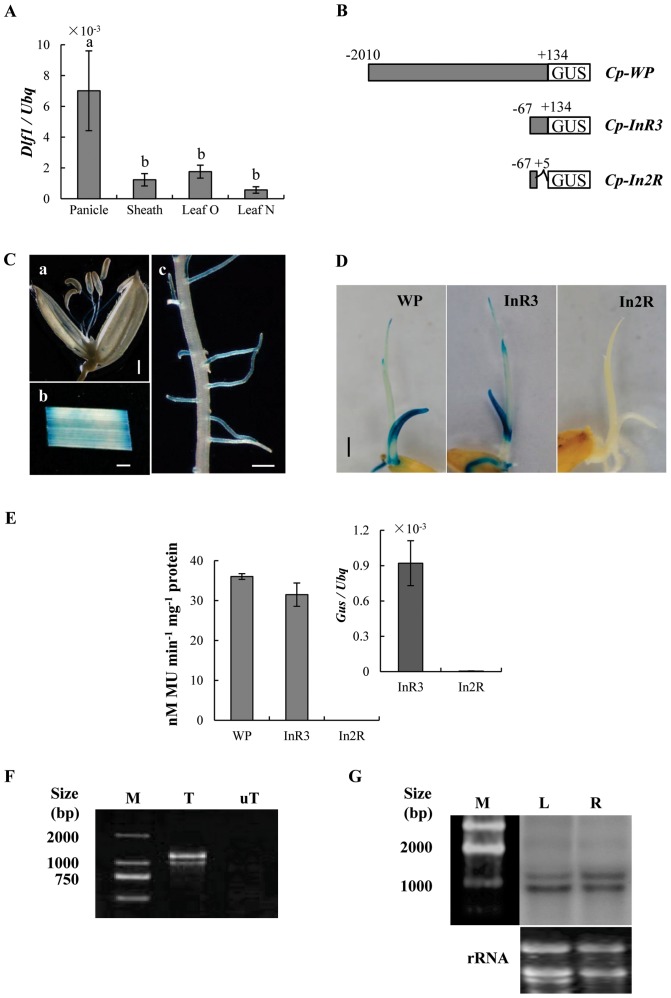
Expression pattern of *Dlf1*. (**A**) Expression of *Dlf1* in different tissues was analyzed by qPCR. RNA was isolated from young panicles, sheathes, older leaves (leaf O) and younger leaves (leaf N). Tissue samples were collected at 4 h after dawn. Values are shown as means ± SD of two biological experiments. Values marked with different letters are significantly different (P<0.05, Duncan test). (**B**) Gus-fused constructs with different lengths of *Dlf1* promoter. (**C**) *Dlf1* promoter-driven *Gus* expression (*Cp-WP*) in (a) young panicles (b) leaves and (c) roots of three-week-old plants. (**D**) Gus histochemical staining of 6-day-old transgenic lines. (**E**) Gus enzyme activity was measured in six-day-old seedlings harboring different constructs. Transcription analysis of *Gus* gene in the transgenic plants. The gene expression was normalized to rice *ubiquitin* gene (*Ubq*) for each sample. Means and their standard deviations are shown from three independent experiments. (**F**) Different transcripts of *Dlf1*. Total RNAs were isolated from leaves of three-week-old ZH11 plants. PCR products were obtained by amplification using templates of reverse-transcribed RNA (T) and RNA (uT) and separated by electrophoresis. (**G**) Northern blot analysis of *Dlf1* expression in leaves (L) and roots (R), using the total RNAs isolated from three-week-old plants. The probes used were the *Dlf1* coding region. rRNA of ethidium bromide staining was used as the loading control. Bar  = 1 mm (**C**) and 2 mm (**D**).

To examine the expression pattern of *Dlf1*, qPCR analysis was performed with total RNA prepared from leaves, sheaths, young panicles and roots of ZH11 wild-type plants grown under natural LD conditions. *Dlf1* was expressed in all tissues examined (Fig. 3A). Further, we stained the transgenic plants harboring the *Dlf1* promoter- driven *Gus* fusion gene (−2010 – +134 bp; *Cp-WP:Gus*, Fig. 3B). Gus staining was observed in leaves, roots and panicles (Fig. 3C), which confirmed the qPCR results. Since the plants harboring *Cp-Ins-Dlf* construct showed the *dlf1* phenotypes, the fragment from the insertion site (−67 – +134 bp; *Cp-InR3:Gus*) of *Dlf1* was also fused with *Gus* gene. Surprisingly, *Cp-InR3:Gus* plants exhibited the similar level of expression as the *Cp-WP:Gus* plants in the young seedlings (Figs. 3D and E) though the *InR3* fragment was a region transcribed. However, the expression of *Gus* gene and Gus activity were low in the *Cp-In2R:Gus* (−67 – +5 bp) transgenic plants, which revealed that the deduced translation region in *InR3* was required for the promoter activity in comparison with the *In2R* fragment.

To obtain the *Dlf1* cDNA, we amplified the open reading frame region from total RNA isolated from ZH11 using RT-PCR with the primers W1 and W5. Two amplified products were obtained (Fig. 3F), the longer one (assigned as *OsWRKY11.1*) encoded the deduced 379-aa Dlf1 protein and the shorter sequence (assigned as *OsWRKY11.2*) contained a 161 bp deletion in the third exon causing a premature stop of translation (Fig. S2). The deduced amino acid sequence of OsWRKY11.2 encodes a protein of 270 residues, which still contains the WRKY domain (aa 205–262). Northern blot analysis revealed two hybridization bands using the *Dlf1* coding region, (Fig. 3G), confirming the existence of alternative splicing of the *Dlf1* mRNAs.

### Dlf1 has transcriptional activation activity in yeast

**Figure 4:**
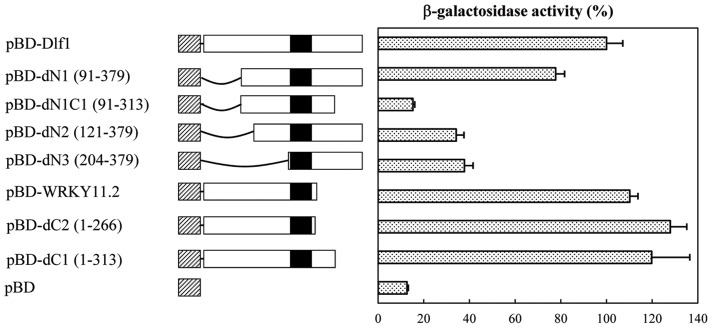
Dlf1 is transactivator in yeast. The full encoding sequence and deletion mutants of *Dlf1* were fused in frame to the *GAL4*-binding domain (BD) in *pBDKT7* to generate various vectors for yeast transformation. The constructed vectors were transformed into yeast AH109 strain, and grew on the selective medium at 30°C for 3 d. The β-galactosidase activity of transformants was determined using *o*-nitrophenyl β-D-galatopyranoside as a substrate. An empty vector *pGBKT7* (*pBD*) was used as the negative control. The values from three independent experiments were shown as means ± SD. Slash boxes represent BD in *pGBKT7* and the black boxes for the WRKY domain of Dlf1, whereas the white boxes represent the rest part of Dlf1, and the line indicates the deleted region. The numbers in the brackets are the start and end positions of each translation product of Dlf1 in the construct.

Dlf1 contains an acidic N-terminus that may function as a transcriptional activation domain. To investigate this possibility, the coding region of the *Dlf1* full length cDNA and its truncated derivatives were fused in frame to the GAL4 DNA-binding domain in the *pGBKT7* vector. The transactivation activity assay in yeast showed that the region of 91–120 aa was required for its transactivation (Fig. 4). We also determined the activation activity of OsWRKY11.2 (*pBD-WRKY11.2*), showing slight increase in activity compared with full length *Dlf1* transcript (*pBD-WRKY11.1*). This result was confirmed by deletion of the 3′ terminal of full length transcript (*pBD-dC2* construct).

**Figure 5:**
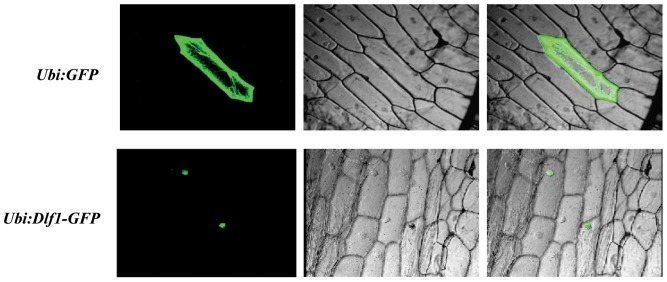
Nuclear localization of Dlf1-GFP fusion protein in onion epidermal cells. Onion epidermal cells were transformed with plasmids expressing GFP (top panel), or the Dlf1-GFP fusion protein (bottom panel) by bombardment and examined after 24 h. GFP fluorescence (left panel) and differential interference contrast image (middle panel) were compared to show the subcellular localization of GFP (cytoplasmic and nuclear) and Dlf1-GFP (nuclear). The images of the right panel were merged for each.

Nuclear localization signal (NLS) of Dlf1 was predicted using cNLS Mapper (http://nls-mapper.iab.keio.ac.jp/). A NLS (amino acid 151–181) was identified with a high score of 7.5. The OsWRKY11.2 protein also contains the sequence of nuclear localization signal. To confirm the subcellular localization of Dlf1, we fused *OsWRKY11.1* with the enhanced green fluorescent protein (*GFP*) gene. The chimeric gene was put under the control of maize ubiquitin (*Ubi*) promoter. The resulting plasmid (*Ubi:Dlf1-GFP*) was then bombarded into onion epidermal cells. Localization of the WRKY11.1-GFP fusion protein was visualized exclusively in the nucleus (Fig. 5), whereas the control GFP (*Ubi:GFP*) was distributed both in the cytoplasm and the nucleus, indicating Dlf1 is a nuclear protein.

### The expression of *Ehd2*, *Ehd1*, *Hd1* and *Hd3a* was repressed in *dlf1* mutant

**Figure 6:**
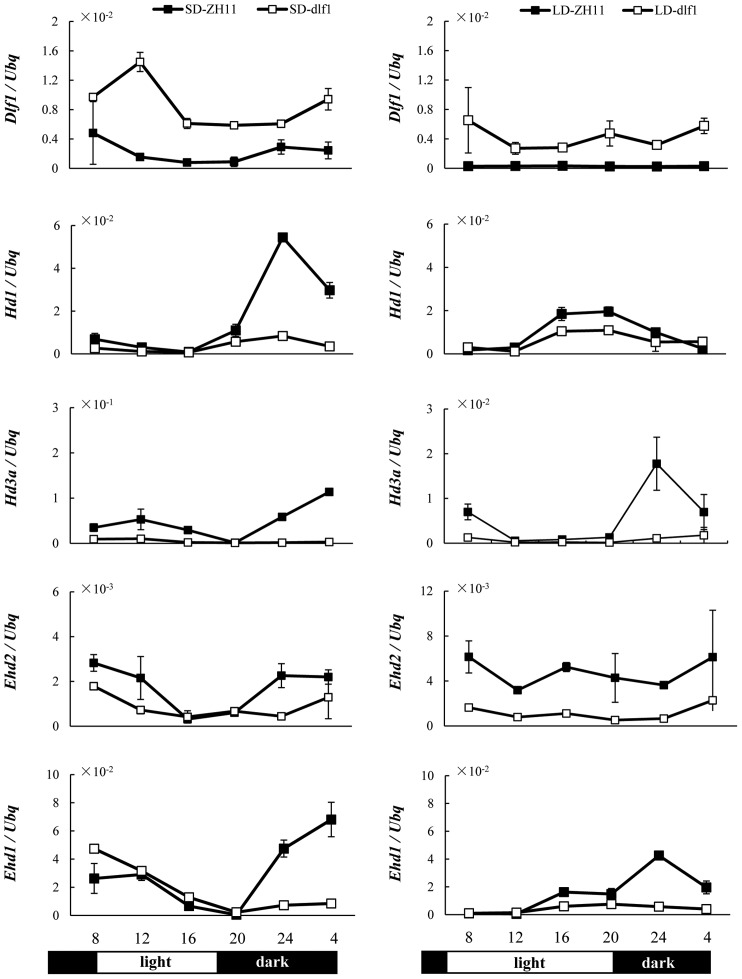
Dlf1 suppresses the expression of *Hd3a*, *Hd1*, *Ehd1* and *Ehd2*. Diurnal expression patterns of *Dlf1*, *Hd1*, *Hd3a, Ehd2* and *Ehd1* in wild-type ZH11 (filled squares) and *dlf1* (open squares) plants under SD (10/14 h light/dark) and LD (14/10 h light/dark) conditions by qPCR analysis. The expression levels are relative to the *ubiquitin* (*Ubq*) mRNA. The plants were grown at conditions as described in Fig. 1. Values are shown as means ± SD of two independent experiments. The open and filled bars at the bottom represent the light and dark periods, respectively.

To determine whether the late flowering phenotype of the *dlf1* mutant was due to the changes in flowering-related gene expression, qPCR analysis was performed in the wild-type and *dlf1* plants. Leaf samples were collected from 40 or 90-d-old plants grown under SD or LD conditions. The developmental stage of the plants was about one month before flowering in ZH11. The total expression levels of *Dlf1* in the mutant were higher than in the wild-type plants under both SD and LD conditions (Fig. 6). Moreover, *Dlf1* mRNA accumulation showed diurnal changes in the wild-type and *dlf1* mutant plants. *Ehd2/RID1/OsId1* is a key regulator in the genetic network that controls photoperiodic flowering in rice, promoting floral transition by upregulating *Ehd1* and then the downstream *Hd3a* [15]–[17]. *Ehd2/RID1/OsId1* mRNA accumulation was reduced in the *dlf1* mutant compared with wild-type plants under SD and LD conditions (Fig. 6). Subsequently, the levels of *Hd3a* and *Ehd1* expression were decreased in the mutant plants. The expression of *Hd1* was also suppressed in the *dlf1* mutant under both SD and LD conditions (Fig. 6). However, the expression of photoperiod-related genes *OsGI*, *Se5* and *Ghd7* were not significantly affected in the *dlf1* mutant (Fig. S3), indicating that Dlf1 specifically suppressed the expression of *Ehd2* and downstream genes.

**Figure 7:**
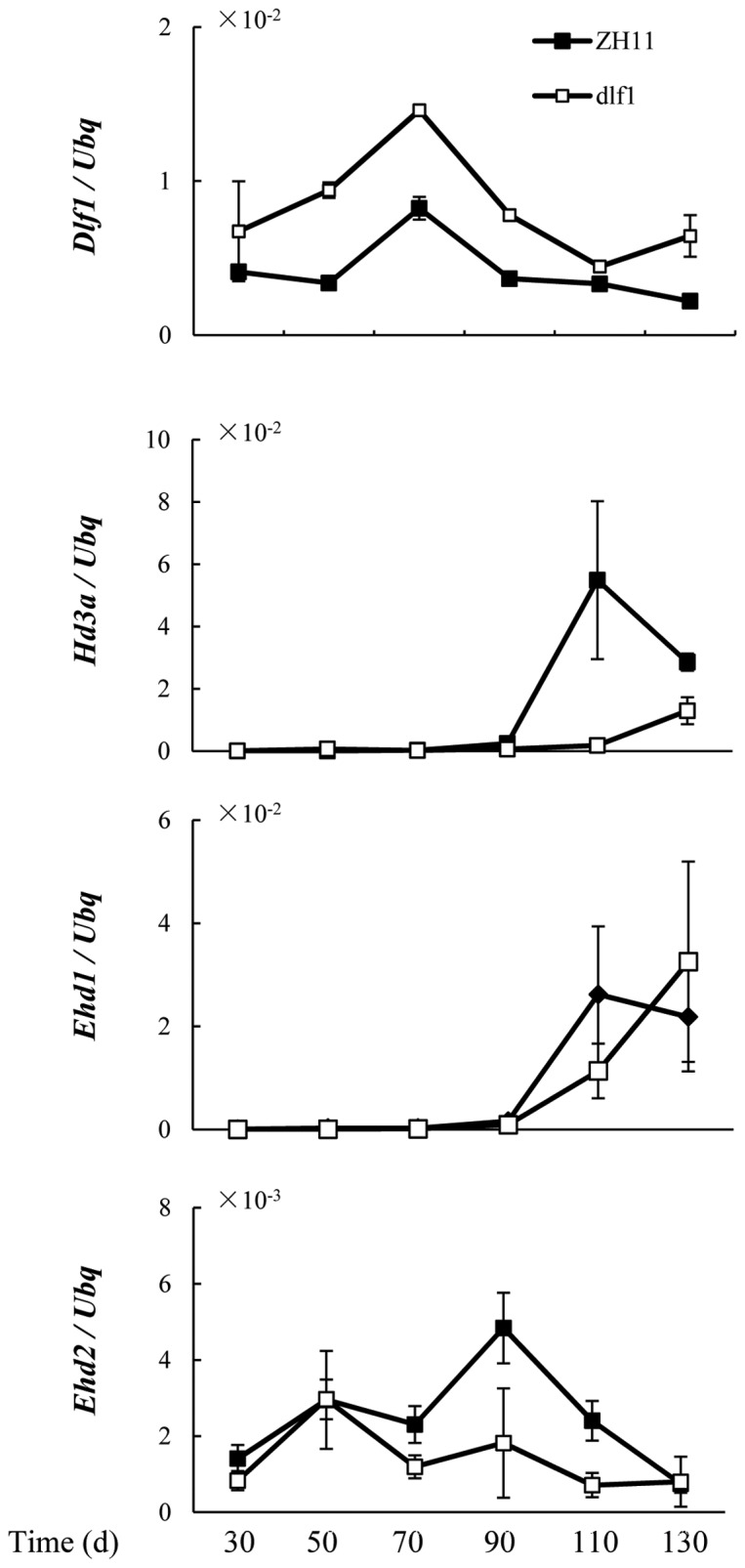
Expression of *Dlf1* and other flowering-time genes during development. Changes of *Dlf1*, *Hd3a*, *Ehd1* and *Ehd2* transcription levels in wild-type (filled squares) and *dlf1* (open squares) plants during development under LD conditions. The expression levels are relative to the *ubiquitin* (*Ubq*) mRNA. The values of *Dlf1* expression in ZH11 were scaled up 10 times. The plants were grown at conditions as described in Fig. 1. Developing leaves were harvested 4 h after dawn. Values are shown as means ± SD of two independent experiments.

Transcription levels of *Dlf1* and the flowering-related genes were also examined at different developmental stages under LD conditions every 20 days. The accumulation of *Dlf1* mRNA slightly increased and reached a peak at 70 d after germination. Subsequently, the transcript level gradually decreased and remained at low levels even after flowering in the wild-type plants (Fig. 7). In the *dlf1* mutant, *Dlf1* transcript accumulated in a pattern quite similar to that of the wild type ZH11 plants, but the expression levels of *Dlf1* were at least 10-fold higher throughout the experiment periods.

### Decrease of *Dlf1* expression showed early flowering under LD conditions

**Figure 8:**
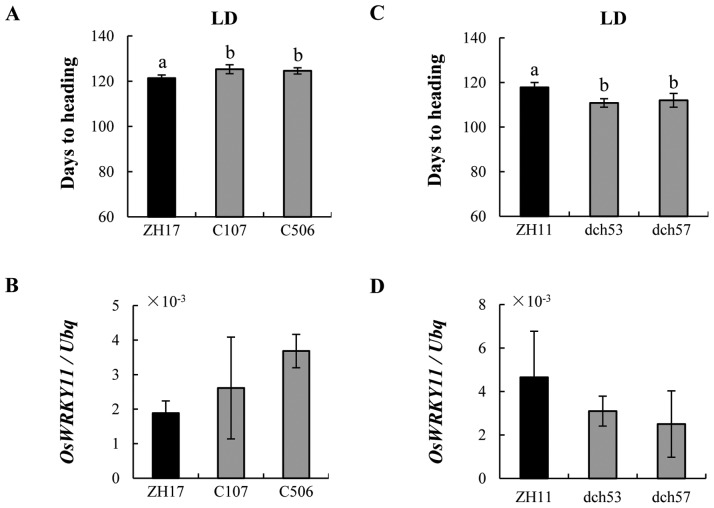
Alternation of *Dlf1* expression changes the heading day of rice. (**A**) The heading time of *Ubi:W11.1* transgenic lines in T_2_ progenies (named as C-) and the ZH17 control under LD conditions. (**B**) Expression of total *OsWRKY11* (including the transferred and endogenous genes) in the *Ubi:W11.1* transgenic and ZH17 plants. (**C**) The heading day of ZH11 and the RNAi lines in T_2_ progenies (named as dch-) under LD condition. (**D**) Expression of total *OsWRKY11* in the RNAi transgenic and ZH11 control plants. Leaves of the first and second youngest were sampled from 90-d old plants. Values are shown as means ± SD of two independent experiments. a and b in figure indicate ranking by Duncan test at P<0.05, starting from a. b is significantly different from a.

To further dissect the function of *Dlf1*, several constructs were generated to examine possible roles of the different transcripts by means of overexpression and RNAi. The full-length *OsWRKY11.1* and the alternatively spliced *OsWRKY11.2* transcript were put under control of the *Ubi* promoter, respectively, generating the *Ubi:W11.1* and *Ubi:W11.2* constructs for rice transformation. To knockdown *Dlf1*, a 276-bp fragment was used for the RNAi construct under the control of the CaMV35S promoter. More than 15 independent overexpressing transgenic lines were obtained for *Ubi:W11.1* in the genetic background of ZH11 and ZH17, respectively. However, only two *Ubi:W11.1* lines of ZH17 genetic background (named as C-) were found to increase *OsWRKY11* expression level and to delay flowering under LD condition comparing with ZH17 plants (Figs. 8A and B). Most of the transgenic lines in ZH11 background (named as OE-) showed slight variations in plant height (Fig. S4B) and insignificant changes in total transcripts of *OsWRKY11* (data not shown). Further, RNAi lines of dch53 and dch57 displayed suppressed transcription of *OsWRKY11* and flowered earlier than the ZH11 controls under LD condition (Figs. 8C and D). These results collectively suggested that *Dlf1* negatively regulate flowering in rice.

### The C-terminus of *Dlf1* plays a role in the regulation of its expression level

**Figure 9:**
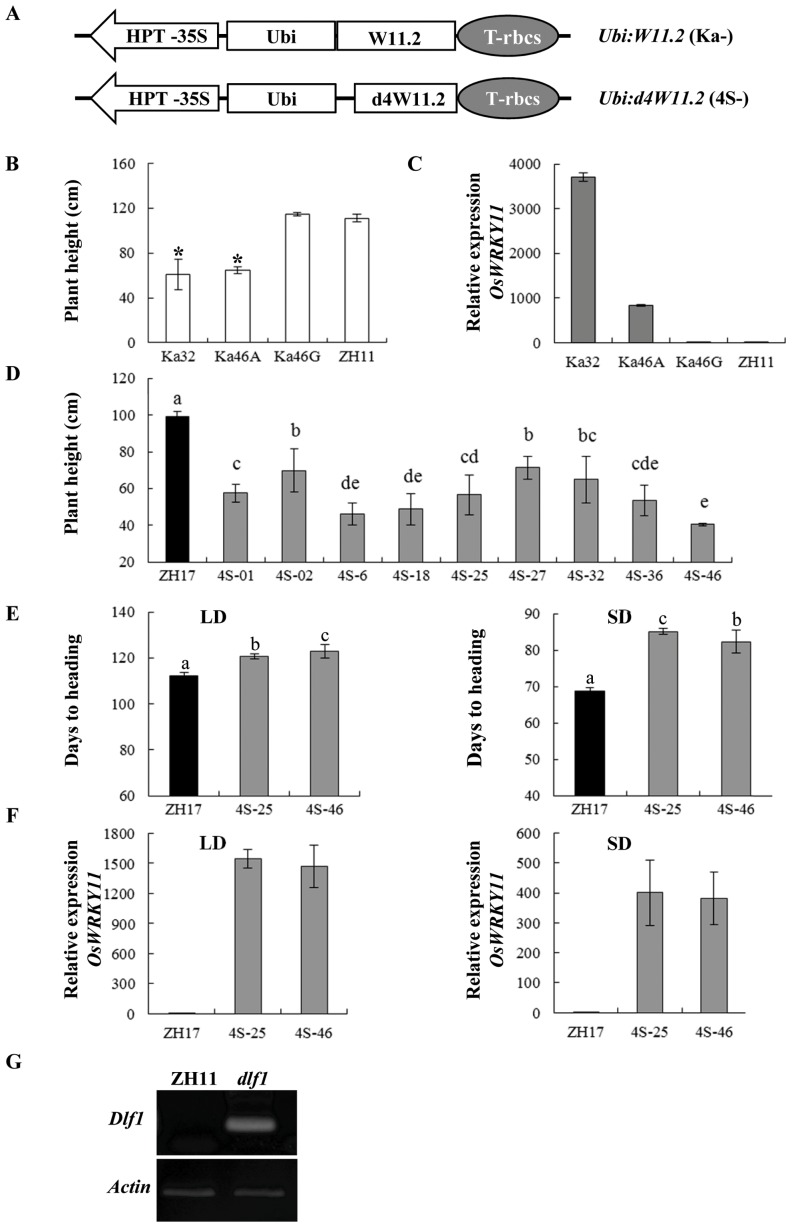
High level expression of the *OsWRKY11.2* leads to dwarfism and late flowering. (**A**) Schematic diagram of *Ubi:W11.2* (Ka-) and *Ubi:d4W11.2* (4S-, with 37-aa deletion at the N-terminus of W11.2) constructs. (**B**) and (**D**) Plant heights of those transformed with *Ubi:W11.2* or *Ubi:d4W11.2* in ZH11 or ZH17 genetic background, respectively. (**C**) Expression of total *OsWRKY11* (including the transferred and endogenous genes) in ZH11 and the *Ubi:W11.2* transgenic lines of T1 progenies under natural LD conditions. The first and second youngest leaves were sampled from 90-d-old plants for RNA isolation. Transcription levels were quantified by qPCR and the gene expression was normalized to rice *ubiquitin* gene (*Ubq*) for each sample. Transcription levels are expressed as ratio to the level of transcript in ZH11. The suffix A for dwarf and G for segregated non-transgenic plants. (**E**) Days to heading of the *Ubi:d4W11.2* plants of T_2_ progenies under SD and LD conditions (the same treatments as in Fig. 6). (**F**) Expression of total *OsWRKY11* in ZH17 and the *Ubi:d4W11.2* transgenic plants of T_2_ progenies under both LD and SD conditions (the same treatments as in Fig. 6). Transcription levels are expressed as ratio to the level of transcript in ZH17. (**G**) Analysis of the possible degraded mRNA of *Dlf1* using the RNA ligase-mediated amplification of 5′ cDNA ends (RLM-RACE). Total RNAs were isolated from the *dlf1* mutant (*dlf1*) and the wild-type (ZH11) of three-week-old plants. Two rounds of PCR were performed: 1) by using an outer primer from the adaptor and a *Dlf1*-specific reverse primer W5; and 2) by using the inner primer from the adaptor and a *Dlf1* gene primer W4 for 30 cycles. *Actin* gene was used as an internal standard. Asterisks indicate significant difference between ZH11 and the overexpression lines (P<0.05, Duncan test). a, b, c, d, and e indicate ranking by Duncan test at P<0.05, starting from a. Different letters indicate significantly difference from each other.

In contrast to *Ubi:W11.1* construct, most of the *Ubi:W11.2* transgenic lines (named as Ka-) exhibited severe dwarfism (Fig. 9B) and delayed flowering of about 2–3 weeks compared with ZH11 under natural LD conditions. The expression of total *OsWRKY11* in transgenic plants was much higher than in ZH11 control and the segregated non-transgenic plants (Fig. 9C), suggesting that the C-terminus of Dlf1 plays a role in controlling its expression level.

Due to the promoter activity of *InR3* fragment, transgenic plants were also obtained with the constructs excluding the N-terminal 37 aa of Dlf1 (assigned as *Ubi:d4W11.1* and *Ubi:d4W11.2*). The *Ubi:d4W11.2* transgenic lines (named as 4S-) presented dwarf and late flowering phenotypes under both LD and SD conditions (Figs. 9D and E), whereas the *Ubi:d4W11.1* plants had no such phenotype (data not shown). Analysis of the expression level of the transgenic plants revealed that the accumulation of *OsWRKY11* total mRNA was increased over 400-fold in the *Ubi:d4W11.2* progenies (Fig. 9F). These results suggest that the C-terminal region of Dlf1 influenced its expression and high level of expression of truncated *OsWRKY11.2* might function in a similar manner as *OsWRKY11.2*.

To get a clue of the effect on *Dlf1* expression, we examined the possible degraded mRNA of *Dlf1* using the RNA ligase-mediated amplification of 5′ cDNA ends (RLM-RACE) [28]. A stronger PCR band was obtained in the *dlf1* mutant than the ZH11 (Fig. 9G). Sequencing verified the degraded positions at 1093 and 1099 bp of *Dlf1*. These observations support the idea that RNA processing proteins or microRNAs may regulate the expression of *Dlf1*, if the level of *Dlf1* mRNA containing the 3′ end reached a limit.

## Discussion

In this study, we demonstrated that the *Dlf1* gene had pleiotropic effects on a variety of traits, including flowering time, plant height, grain number and leaf rolling. Yield, plant height and heading date are the most important agronomic traits in rice, and a number of genes have been isolated that control each of these traits. For example, *Gn1a*, a gene for cytokinin oxidase/dehydrogenase, regulates the number of grains per panicle [5], and a RING-type E3 ligase (*GW2*) controls grain width and weight [6]. The height of a rice plant is regulated by the gibberellin-insensitive gene *Dwarf 1*, encoding the alpha-subunit of GTP-binding protein, and the brassinosteroid biosynthesis gene *Dwarf 11* [1], [3]. *Ghd7*, encoding a CCT-domain protein, was shown to have multiple effects on grain number, heading date and plant height [19]. *DTH8/Ghd8/Hd5*, encoding a HAP3 subunit of the CCAAT-box binding protein, is also reported to suppress rice flowering under LD conditions and regulate plant height and yield potential [23], [24]. Our data indicate that the rice WRKY transcription factor Dlf1 also widely affects rice development. *Dlf1* regulates plant height by altering cell size in the internodes, similar to the effect of *DTH8* but different from that of *Ghd7* (Fig. S1) [19], [23]. The phenotypes of short internode length and leaf rolling in the *dlf1* mutant are supported by a recent report of *OsWRKY11* transgenics, which is controlled by the promoter of heat shock-inducible *HSP101* gene [29]. Among the four transgenic rice plants reported, three had bent leaves or dwarf phenotype, and two had significantly enhanced heat and drought tolerance under heat induction conditions.

The T-DNA insertion site in the *dlf1* mutant was 67 bp upstream of the predicted translational starting site of *OsWRKY11* (Fig. 2A). However, the *Dlf1* expression was significantly increased in comparison with the wild-type plants. Transgenic plants harboring the genomic DNA of *Dlf1*, starting from the T-DNA insertion site to the end of *Dlf1* coding region, recapitulated the *dlf1* phenotypes (Fig. 2C), suggesting that the region has promoter function. This is confirmed by fusion with the *Gus* reporter gene (Fig. 3B, D and E). However, the Gus activities of *Cp-WP:Gus* and *Cp-InR3:Gus* were at a similar level, inconsistent with a higher level of expression *Dlf1* in the *dlf1* mutant than that of the wild-type, implying a suppressor element existed outside of the *WP* fragment used in the experiment. We also generated *Dlf1* overexpressing and RNAi transgenic lines. Unexpectedly, most of the *OsWRKY11.1* transgenic plants did not show morphological differences to controls (Fig. S4). Nevertheless, two RNAi lines with decrease in *OsWRKY11* expression showed early flowering under LD conditions (Fig. 8C and D). On the other hand, the accumulation of total *OsWRKY11* mRNA (including the endogenous and transferred gene) was extremely high in the lines harboring the *Ubi:W11.2* construct, which is 109 aa shorter than *Ubi:W11.1* in the C-terminus (Fig. S2). These results suggested that the C-terminus of *Dlf1* was involved in controlling accumulation level of its mRNA. This notion is further supported by comparison of the transgenic plants containing *Ubi:d4W11.1* or *Ubi:d4W11.2* constructs. Interestingly, most of the *Ubi:W11.2* and *Ubi:d4W11.2* transgenic plants exhibited dwarf and late flowering phenotypes, similar to the *dlf1* mutant (Fig. 9). Likely, OsWRKY11.2 retained the transactivation activity (Fig. 4) and the sequence of nuclear localization signal (position 179–187 aa). The results suggested that the high level of OsWRKY11.2, or its N-terminus-truncated protein might function as a negative regulator. This information also implies that different splicesomes of *Dlf1* might work together to regulate downstream gene expression, although further study is required to test the existence of alternative splicing *in planta*.

RNA processing plays an important role in control of plant flowering time. FLOWERING LOCUS C (FLC), a repressor of the transition to flowering in *Arabidopsis*, functions to delay flowering by down-regulating expression of genes promoting the floral state. Processing of *FLC* mRNA is regulated by autonomous pathway components of FCA and FY, which encode a pre-mRNA processing protein and a homolog of the yeast RNA 3′-processing factor Pfs2p, respectively [30], [31]. *FCA* expression is also regulated through alternative processing of its pre-mRNA into four different transcripts, in which only the fully spliced *FCA* transcripts can promote flowering [30]. Furthermore, *FCA* negatively regulates its own expression by increasing cleavage and polyadenylation within intron 3, thus limiting the production of active FCA protein to keep the balance of pathways controlling flowering time. They also found that active *FCA* can be overexpressed only when the *cis*-element within intron 3 required for the negative feedback is removed [32], [33]. As mentioned above, the accumulation of *OsWRKY11.1* mRNA was not much increased in the overexpressing plants. An explanation is that the C-terminal part of OsWRKY11.1 interacts with a protein involved in regulation of the *OsWRKY11.1* mRNA level. When the OsWRKY11.1 protein reaches a threshold level it will activate the protein–protein interaction and decrease accumulation of the *OsWRKY11.1* transcript. This or another interaction might also possibly involve in the alternative splicing of *Dlf1*.

Rice is a facultative SD plant which flowers under LD conditions. As a counterpart of the GI–CO–FT signaling pathway in *Arabidopsis*, the rice orthologous proteins consist of OsGI–Hd1–Hd3a. The clock-associated protein OsGI upregulates *Hd1* expression and in turn Hd1 induces the expression of *Hd3a* during SD conditions in rice [9], [12], [13]. The expression of *Hd3a* is also induced by the Ehd1 activator, which is an evolutionarily unique gene in rice with no counterpart in *Arabidopsis* [15]. Ehd2/RID1/OsId1 was found to promote flowering under both SD and LD conditions by upregulating *Ehd1* expression [16]–[18]. Since *Ehd2/RID1/OsId1* expression was suppressed under both SD and LD conditions in the *dlf1* mutant (Fig. 6), the late flowering phenotype of the mutant is easily explained by loss of the promoting action of *Ehd2/RID1/OsId1* (Fig. 9).

*Dlf1* was expressed in leaves, roots and panicles (Fig. 3A). Expression of *Dlf1* in leaves is consistent with the role of genes in flowering-time regulation, such as *Ehd2* and *Ghd7* [16], [19]. *Ehd2/RID1/OsId1* is considered the master switch for the transition from vegetative to reproductive phase, a crucial process in higher plants. We found that increased *Dlf1* expression delayed the phase transition and initiation of floral induction, leading to late flowering in the *dlf1* mutant. This was further supported by studies of gene expression in the whole developmental process, which showed that *Ehd2/RID1/OsId1* is suppressed in the *dlf1* mutant with a higher level of *Dlf1* mRNA accumulation under LD conditions (Fig. 7). Diurnal expression of *Dlf1* was observed under both LD and SD conditions; however, the expression of photoperiod-related genes *OsGI* and *Se5* was not significantly changed between the mutant and wild-type plants (Fig. 6; Fig. S3), suggesting that *Dlf1* is unlikely to be upstream of these genes in the pathways of photoperiodic flowering in rice. WRKY proteins are a super family of plant transcription factors, which are characterized by binding specifically with W-box (a core sequence of TGAC). Genetic and molecular analyses have revealed that *WRKY* genes play roles in diverse biotic and abiotic stresses, as well as in development [34]–[36]. It is commonly observed that a single transcription factor may regulate multiple plant processes and that some may work in a redundant manner. AtWRKY6 is reported to be associated with senescence- and defense-related processes, and was shown to respond to low Pi stress in *Arabidopsis* [37], [38]. Recently, Roboni et al. [39] have shown that GI plays a key role in photoperiodic cues and drought escape response via ABA-dependent activation of florigens and SOC1. The *gi* mutants exhibit hyper tolerance to oxidative stress, enhanced salt tolerance, and elevated starch content, highlighting the importance of carbohydrate metabolism in the regulation of flowering [40]–[43]. Meanwhile, SOC1 and FLC are mediated in crosstalk between cold response and flowering time regulation [44].

Our data combined with others indicate that Dlf1 play important roles on plant height, heading date, yield potential and responses to abiotic stress [29]. The CCT-domain protein Ghd7 and DTH8 protein have been demonstrated to have pleiotropic effects on heading time, height and yield potential [19], [23]. Further investigation of their relationship should help illuminate the complexities of these important agronomic traits, as well as aid in manipulation of the traits for yield improvement.

## Materials and Methods

### Plant materials and growth conditions

Rice seeds of the wild-type (*Oryza sativa* L. *japonica*, Zhonghua 11 or Zhonghua 17, ZH11 or ZH17), mutant and transgenic progenies were sterilized and germinated on half-strength Murashige and Skoog medium for 7 d and then transferred to soil in a greenhouse. For flowering time measurements, plants were grown either in 10/14 h light/dark for SD or 14/10 h light/dark for LD. Rice flowering time was measured in days from germination until emergence of the first panicle. For diurnal expression analyses, young leaves were harvested from wild-type ZH11 and the *dlf1* mutant of 40-d-old (SD) or 90-d-old (LD) plants at 4-h intervals for a total of 24 h. To analyze gene expression during development, the first and second youngest leaves from three plants were harvested from 30, 50, 70, 90, 110 and 130-d-old plants at 4 h after dawn under LD condition. The rice plants examined under natural field conditions were sown at late April and transplanted at early June in the experimental field of China Agricultural University, Beijing (39°54′N, 116°23′E), China.

### Genotyping of mutant plants and Tail-PCR

For genotyping analysis, the *dlf1* segregating population was assayed by PCR using the primers of PddSalF, TR1 and W6 (Table S1). The primer pair of TR1 and W6 was used for amplification of the T-DNA insertion. Thermal asymmetric interlaced PCR (Tail-PCR) method was used to isolate genomic fragment flanking of the T-DNA insertion site from the *dlf1* mutant plant. The primers TR1, TR2, TR3, AD1, AD2 and AD3 are shown in Table S1.

### Vector construction and transformation

The full-length coding region of *Dlf1* was obtained by PCR amplification using the primers W1 and W5, along with a shorter product (assigned as *OsWRKY11.2*). The *OsWRKY11.1* and *OsWRKY11.2* cDNAs were put under the control of a maize ubiquitin promoter in a modified *pCambia 1301* vector to generate *Ubi:W11.1* and *Ubi:W11.2* for overexpression [34]. Similarly, the PCR products, amplified with the primer pairs of d40BIF/W10H3r and d40BIF/W10SH3r were used to construct *Ubi:d4W11.1* and *Ubi:d4W11.2* vectors, with deletions of the 5′-ends in comparison with *OsWRKY11.1* and *OsWRKY11.2*, respectively. Each overexpressing construct contained a Flag tag in the 5′-end of the gene. The *Dlf1* fragment of 276 bp (from −39 to +237 bp) was used to generate the *Dlf1*-RNAi plasmid using procedures similar to the previous description [34]. The hairpin structure was put under the control of the CaMV35S promoter (*Cam35S:dsW11*). For promoter constructs, the PCR products were fused to *β-glucuronidase* (*Gus*) reporter gene as following: the *Dlf1* promoter from −2010 to +134 bp as *Cp-WP:Gus*, −67 – +134 bp as *Cp-InR3:Gus*, and −67 – +5 bp as *Cp-In2R:Gus*. For complementation, the genomic DNA fragment from −67 to the end of *Dlf1* was put into a modified *pCambia 1301* vector, designed as *Cp-Ins-Dlf*. All vectors were verified by sequencing and transformed into rice through *Agrobacterium*-mediated transformation [35]. The transgenic lines obtained were determined by PCR amplifications.

### Transactivation activity assay

The coding region of *Dlf1* was amplified with the primers W10EI and W10BgSal (Table S3). The PCR product was fused to the GAL4 DNA binding-domain vector *pGBKT7* (*pBD*, Clotech) to generate the plasmid *pBD–Dlf1*. Similarly, a fragment of *Dlf1* encoding amino acids 91–379 (*pBD–dN1*), 121–379 (*pBD–dN2*), 204–379 (*pBD–dN3*), 91–313 (*pBD–dN1C1*), the first 313 amino acids (*pBD–dC1*), the first 266 amino acids (*pBD–dC2*), or OsWRKY11.2 (*pBD-WRKY11.2*) were fused to the GAL4 DNA binding-domain. These constructs or empty vector *pBD* were individually transformed into yeast cells of AH109 strain and grown on SD–Trp selective medium at 30°C for 3 d. An assay of β-galactosidase activity was performed with transformed cell lines grown in liquid SD-Trp medium using *o* -nitrophenyl β-D-galactopyranoside as a substrate, according to the manufacturer's protocol.

### Subcellular localization of Dlf1

The coding sequence of *Dlf1* was amplified and fused in frame to the upstream of green fluorescent protein (*GFP*) gene to generate the *CamUbi:Dlf1-GFP* construct. The resultant and the control *CamUbi:GFP* vectors were transformed into onion (*Allium cepa*) inner epidermal cells by bombardment using the PDS-1000/He system (Bio-Rad) with DNA-coated gold particles. The transformed cells were cultured on 1/2 MS medium at 28°C for 2 d and observed under a confocal microscope (Bio-Rad MRC 1024).

### Gus assay and histochemical staining

Gus activity assay and histochemical staining were performed as described [45] and photographed using a Nikon SMZ 1000 stereoscope with a Nikon digital sight DS-SM camera or Nikon camera.

### RNA gel-blot

Trizol reagent was used to extract the RNA from rice tissues. Total RNA was fractionated in 1.5% agarose containing formaldehyde, blotted onto Hybond-N^+^ nylon membrane and probed with the coding sequence of *Dlf1*. Hybridization was performed as previously described [34] and the membrane was autoradiographed by using a phosphoimaging system (Amersham Pharmacia Biotech, UK).

### Real-time quantitative RT-PCR

Total RNA was isolated from different rice tissues using Trizol reagent following the manufacturer's procedures. DNaseI-treated RNAs were reverse transcribed with oligo (dT) and random primers. Real-time quantitative RT-PCR (qPCR) was performed in a final volume of 20 µL, including 10 µL SYBR Premix EX Taq (Takara), 2 µL of the diluted first-strand cDNA as templates and 0.2 µM of each primer. The reactions were carried out with an ABI PRISM 7000 or ABI StepOne in the following program: 95°C for 2 min, 40 cycles of 95°C for 5 s, 60°C for 20 s, and 72°C for 31 s. Every experiment was repeated more than twice. The primers of *Ehd2*, *Hd1*, *Ehd1*, *Hd3a*, *Ubq*, *Ghd7*, *OsGI*, *Se5 FTL6* and *Dlf1* are listed in Table S2. The level of *ubiquitin* (*Ubq*) expression was used to normalize the expression ratio of each gene.

### RLM-RACE PCR

The RNA ligase-mediated amplification of 5′ cDNA ends (RLM-RACE) was performed according to the manufacturer's manual with modifications. Two µg total RNA was directly ligated to a RNA adaptor using T4 RNA ligase and transcribed by random primers. PCR was performed by using an outer primer from the adaptor and a *Dlf1*-specific reverse primer W5 at conditions of 95°C for 3 min (1 cycle), 95°C for 30 s, 55°C for 30 s, 72°C for 1 min (20 cycles), then 72°C for 10 min (1 cycle). A second round of nested PCR was amplified by using the inner primer and a *Dlf1* gene primer W4 for 30 cycles. *Actin* gene was used as an internal standard. The RACE PCR products were sequenced to identify the mRNA degraded ends.

## Supporting Information

Figure S1 **Phenotypes of Zhonghua 11 (ZH11) and** ***dlf1*** **mutant.** (**A**) Longitudinal section of the stems approximately 2 cm above the upper-most nodes from the tiller culms of plants. (**B**) Number of spikelets per panicle. Values are means ± SD, n = 20. (**C**) 1000-grain weight. Values are means ± SD, n = 10. (**D**) Leaf rolling. (**E**) Transverse sections of the middle part of the first leaf from tillering plants. The plants were grown in the experimental field under natural LD conditions.
(PDF)

Figure S2 **Multiple sequence alignment of** ***OsWRKY11*** **cDNAs and proteins.** (**A**) The full-length cDNA of *Dlf1* (*OsWRKY11.1*) and the alternatively spliced transcript *OsWRKY11.2* were aligned using the CLUSTAL W program. (B) The alignment of Dlf1 and OsWRKY11.2 proteins using CLUSTAL W program.
(PDF)

Figure S3 **Expression of** ***OsGI*** **,** ***Ghd7, Se5*** **, and** ***FTL6*** **.** Diurnal expression patterns of *OsGI, Ghd7, Se5*, and *FTL6* in the ZH11 control (filled circle) and the mutant *dlf1* (open circle) plants under SD (10 h light/14 h dark) and LD (14 h light/10 h dark) conditions by qPCR analysis. The expression levels are relative to the *ubiquitin* (*Ubq*) mRNA. Values are shown as means ± SD of two independent experiments. The open and filled bars at the bottom represent the light and dark periods, respectively.
(PDF)

Figure S4 **Heights of** ***Ubi:W11.1*** **transgenic plants.** (**A**) Schematic diagram of *Ubi:W11.1* construct (OE-). (**B**) Plant heights of some *Ubi:W11.1* lines in T_2_ progenies and the ZH11 control. Values are means ± SD.
(PDF)

Table S1 **Primers of** ***Dlf1*** **for genotype, expression, and vector construction.**
(DOCX)

Table S2 **Primers of photoperiod- and flowering-time-related genes for real-time RT-PCR.**
(DOCX)

Table S3 **Primers for transactivation activity.**
(DOCX)
