# Supplementary material for: Dlf1, a WRKY Transcription Factor, Is Involved in the Control of Flowering Time and Plant Height in Rice
Source: PLoS One. 2014 Jul 18;9(7):e102529. doi: 10.1371/journal.pone.0102529 (PMC4103817; doi:10.1371/journal.pone.0102529)
Supplement: Figure S1 — Phenotypes of Zhonghua 11 (ZH11) and dlf1 mutant. (A) Longitudinal section of the stems approximately 2 cm above the upper-most nodes from the tiller culms of plants. (B) Number of spikelets per panicle. Values are means ± SD, n = 20. (C) 1000-grain weight. Values are means ± SD, n = 10. (D) Leaf rolling. (E) Transverse sections of the middle part of the first leaf from tillering plants. The plants were grown in the experimental field under natural LD conditions. (PDF) [file pone.0102529.s001.pdf]

(A) Cell size

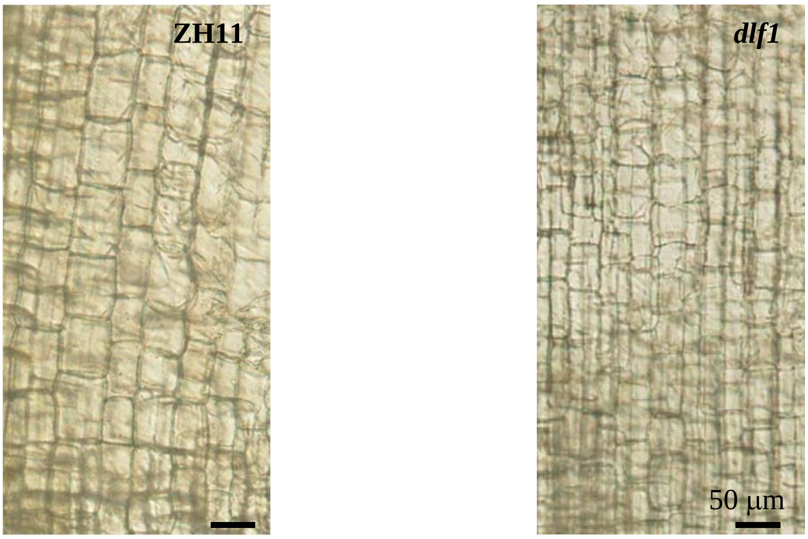

(82.5±9.35 µm) × (55.8±2.04 µm) (59.5±5.99 µm) × (46±4.59 µm)

(B) Number of spikelets per panicle

Zhonghua11: 294.9 ± 13.3 ; *dlf1* mutant: 189.3 ± 22.3

(C) 1000-grain weight

Zhonghua11: 28.83 ± 0.12 g ; *dlf1* mutant: 20.13 ± 0.17 g

(D)

ZH11 **_dlf1_**

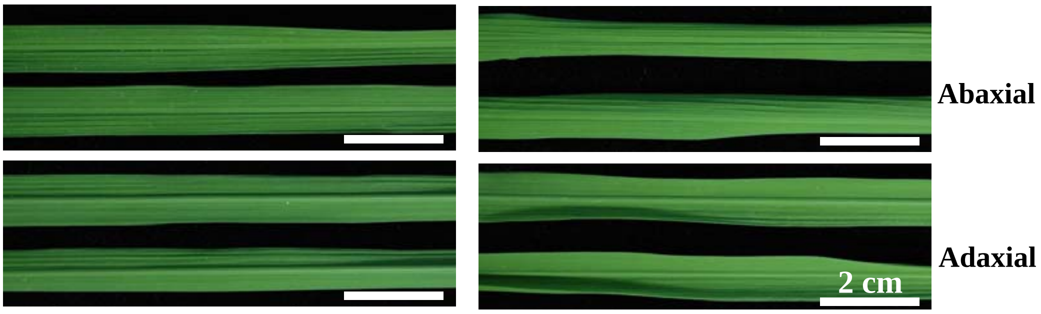

(E)

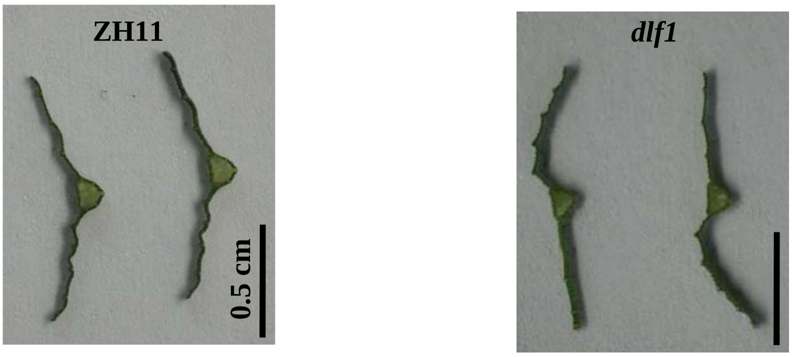

Supplemental Figure S1
