# Supplementary material for: Dlf1, a WRKY Transcription Factor, Is Involved in the Control of Flowering Time and Plant Height in Rice
Source: PLoS One. 2014 Jul 18;9(7):e102529. doi: 10.1371/journal.pone.0102529 (PMC4103817; doi:10.1371/journal.pone.0102529)
Supplement: Figure S2 — Multiple sequence alignment of OsWRKY11 cDNAs and proteins. (A) The full-length cDNA of Dlf1 (OsWRKY11.1) and the alternatively spliced transcript OsWRKY11.2 were aligned using the CLUSTAL W program. (B) The alignment of Dlf1 and OsWRKY11.2 proteins using CLUSTAL W program. (PDF) [file pone.0102529.s002.pdf]

Supplemental Figure S2

(A)
CLUSTAL W (1.83) multiple sequence alignment of cDNAs

```
Dlf1          ATGTCTTCTG GAGGAGGAGG AGGAGGAGGA GGAGATCGCC ATGGCCCCTA
OsWRKY11.2    ATGTCTTCTG GAGGAGGAGG AGGAGGAGGA GGAGATCGCC ATGGCCCCTA

Dlf1          CCACCAGCAC GGGCACCTCG GCCGCGGCGA AGGCGCTGAT TACGTGTACA
OsWRKY11.2    CCACCAGCAC GGGCACCTCG GCCGCGGCGA AGGCGCTGAT TACGTGTACA

Dlf1          GCAGCAGCGA CATGGAGAGC TTCTTCTTCA GCCAACCCGG GGGCGTCGGC
OsWRKY11.2    GCAGCAGCGA CATGGAGAGC TTCTTCTTCA GCCAACCCGG GGGCGTCGGC

Dlf1          ATCGGCGGGG GTGGTGGTGG CGTCGTCGGC GCCGGCGGTG CCGACGAGAT
OsWRKY11.2    ATCGGCGGGG GTGGTGGTGG CGTCGTCGGC GCCGGCGGTG CCGACGAGAT

Dlf1          CATGCCGTAC TCCAGCATCA CGGACTACCT GCAGGGGTTA TTGGACCCCT
OsWRKY11.2    CATGCCGTAC TCCAGCATCA CGGACTACCT GCAGGGGTTA TTGGACCCCT

Dlf1          CCGGGCTAGC TCGGCACCTC GACGTGGCGT GCCCGTCGTC TCAGGACACG
OsWRKY11.2    CCGGGCTAGC TCGGCACCTC GACGTGGCGT GCCCGTCGTC TCAGGACACG

Dlf1          GTGGTCAAGC AGGAGCTGTC GGTGGATGTG ACGAGCCACG ACAGCCAGGG
OsWRKY11.2    GTGGTCAAGC AGGAGCTGTC GGTGGATGTG ACGAGCCACG ACAGCCAGGG

Dlf1          CACCGGCGGC GTCGCCGGAG AAGGCGTCGC GCAGGCCACG CCGAACTCGT
OsWRKY11.2    CACCGGCGGC GTCGCCGGAG AAGGCGTCGC GCAGGCCACG CCGAACTCGT

Dlf1          CGGCGTCCTT CTCGTCCAGC GACGGAGAGG CGGAGGGCGG CAAATCGTCA
OsWRKY11.2    CGGCGTCCTT CTCGTCCAGC GACGGAGAGG CGGAGGGCGG CAAATCGTCA

Dlf1          CGCCGGTGCA AGAAGGGTCA GGCGAAGGCG GAGGAGGAGG ACGACAAGGA
OsWRKY11.2    CGCCGGTGCA AGAAGGGTCA GGCGAAGGCG GAGGAGGAGG ACGACAAGGA

Dlf1          TGAGGAGGAT GGAGAGAACT CCAAGAAACC GAACAAGCCC AAGAAGAAAG
OsWRKY11.2    TGAGGAGGAT GGAGAGAACT CCAAGAAACC GAACAAGCCC AAGAAGAAAG

Dlf1          CTGAGAAGAG GCAGCGGCAG CCTCGCGTGG CGTTCCTCAC CAAGAGCGAG
OsWRKY11.2    CTGAGAAGAG GCAGCGGCAG CCTCGCGTGG CGTTCCTCAC CAAGAGCGAG

Dlf1          GTTGATCACC TCGAGGACGG CTACCGGTGG CGCAAGTACG GCCAGAAGGC
OsWRKY11.2    GTTGATCACC TCGAGGACGG CTACCGGTGG CGCAAGTACG GCCAGAAGGC

Dlf1          AGTCAAGAAC AGCCCATACC CGAGGAGCTA CTACCGGTGC ACGACGCCCA
OsWRKY11.2    AGTCAAGAAC AGCCCATACC CGAGGAGCTA CTACCGGTGC ACGACGCCCA

Dlf1          AGTGCGGTGT GAAGAAGCGG GTGGAGCGGT CGTACCAGGA CCCCTCCACG
OsWRKY11.2    AGTGCGGTGT GAAGAAGCGG GTGGAGCGGT CGTACCAGGA CCCCTCCACG

Dlf1          GTGATCACCA CGTACGAAGG GCAGCACACG CACCACAGCC CGGCGAGCCT
OsWRKY11.2    GTGATCACCA CGTACGAAGG GCAGCACACG CACCACAGCC CGGCGAGCC-

Dlf1          CCGCGGCGGC GGCGGCGGCG TCGGCATCGT CGGAGGGCAC CACCACCACC
OsWRKY11.2    ---------- ---------- ---------- ---------- ----------

Dlf1          ACCTCTTCAT GCCCGGCGTG CATGGCCTGC CGCCGTCGCA CCTCATGCCG
OsWRKY11.2    ---------- ---------- ---------- ---------- ----------

Dlf1          GCGGGGTTCC ACCCTGAGCT GATGGGCCTC ATGCACCACC ACCCGGCCAT
OsWRKY11.2    ---------- ---------- ---------- ---------- ----------

Dlf1          GGCCGCCGCC GCCGCAAACC CTAGCATGTA CTTCCCGGGC GTAGCTGCTT
OsWRKY11.2    ---------- GCCGCAAACC CTAGCATGTA CTTCCCGGGC GTAGCTGCTT

Dlf1          CTGCTCCGCC GCCTCCTGCT GTGGCTGGCG GCGGTGCAAT GCCGCCCAAC
OsWRKY11.2    CTGCTCCGCC GCCTCCTGCT GTGGCTGGCG GCGGTGCAAT GCCGCCCAAC

Dlf1          GATCATCCTC CTCTTCAGCA GCATCACTTC ACTGACTACG CCCTGCTGCA
OsWRKY11.2    GATCATCCTC CTCTTCAGCA GCATCACTTC ACTGACTACG CCCTGCTGCA

Dlf1          AGACCTTTTC CCTTCCACAA TGCCCAGCAG CAACCCATGA
OsWRKY11.2    AGACCTTTTC CCTTCCACAA TGCCCAGCAG CAACCCATGA
```

```
 (B)
CLUSTAL W (1.83) multiple sequence alignment of proteins


Dlf1          MSSGGGGGGG GDRHGPYHQH GHLGRGEGAD YVYSSSDMES FFFSQPGGVG
OsWRKY11.2    MSSGGGGGGG GDRHGPYHQH GHLGRGEGAD YVYSSSDMES FFFSQPGGVG

Dlf1          IGGGGGGVVG AGGADEIMPY SSITDYLQGL LDPSGLARHL DVACPSSQDT
OsWRKY11.2    IGGGGGGVVG AGGADEIMPY SSITDYLQGL LDPSGLARHL DVACPSSQDT

Dlf1          VVKQELSVDV TSHDSQGTGG VAGEGVAQAT PNSSASFSSS DGEAEGGKSS
OsWRKY11.2    VVKQELSVDV TSHDSQGTGG VAGEGVAQAT PNSSASFSSS DGEAEGGKSS

Dlf1          RRCKKGQAKA EEEDDKDEED GENSKKPNKP KKKAEKRQRQ PRVAFLTKSE
OsWRKY11.2    RRCKKGQAKA EEEDDKDEED GENSKKPNKP KKKAEKRQRQ PRVAFLTKSE

Dlf1          VDHLEDGYRW RKYGQKAVKN SPYPRSYYRC TTPKCGVKKR VERSYQDPST
OsWRKY11.2    VDHLEDGYRW RKYGQKAVKN SPYPRSYYRC TTPKCGVKKR VERSYQDPST

Dlf1          VITTYEGQHT HHSPASLRGG GGGVGIVGGH HHHHLFMPGV HGLPPSHLMP
OsWRKY11.2    VITTYEGQHT HHSPASRRKP ---------- ---------- ----------

Dlf1          AGFHPELMGL MHHHPAMAAA AANPSMYFPG VAASAPPPPA VAGGGAMPPN
OsWRKY11.2    ---------- ---------- ---------- ---------- ----------

Dlf1          DHPPLQQHHF TDYALLQDLF PSTMPSSNP
OsWRKY11.2    ---------- ---------- ---------
```
