# Supplementary figures and images for: Dlf1, a WRKY Transcription Factor, Is Involved in the Control of Flowering Time and Plant Height in Rice
Source: PLoS One. 2014 Jul 18;9(7):e102529. doi: 10.1371/journal.pone.0102529 (PMC4103817; doi:10.1371/journal.pone.0102529)

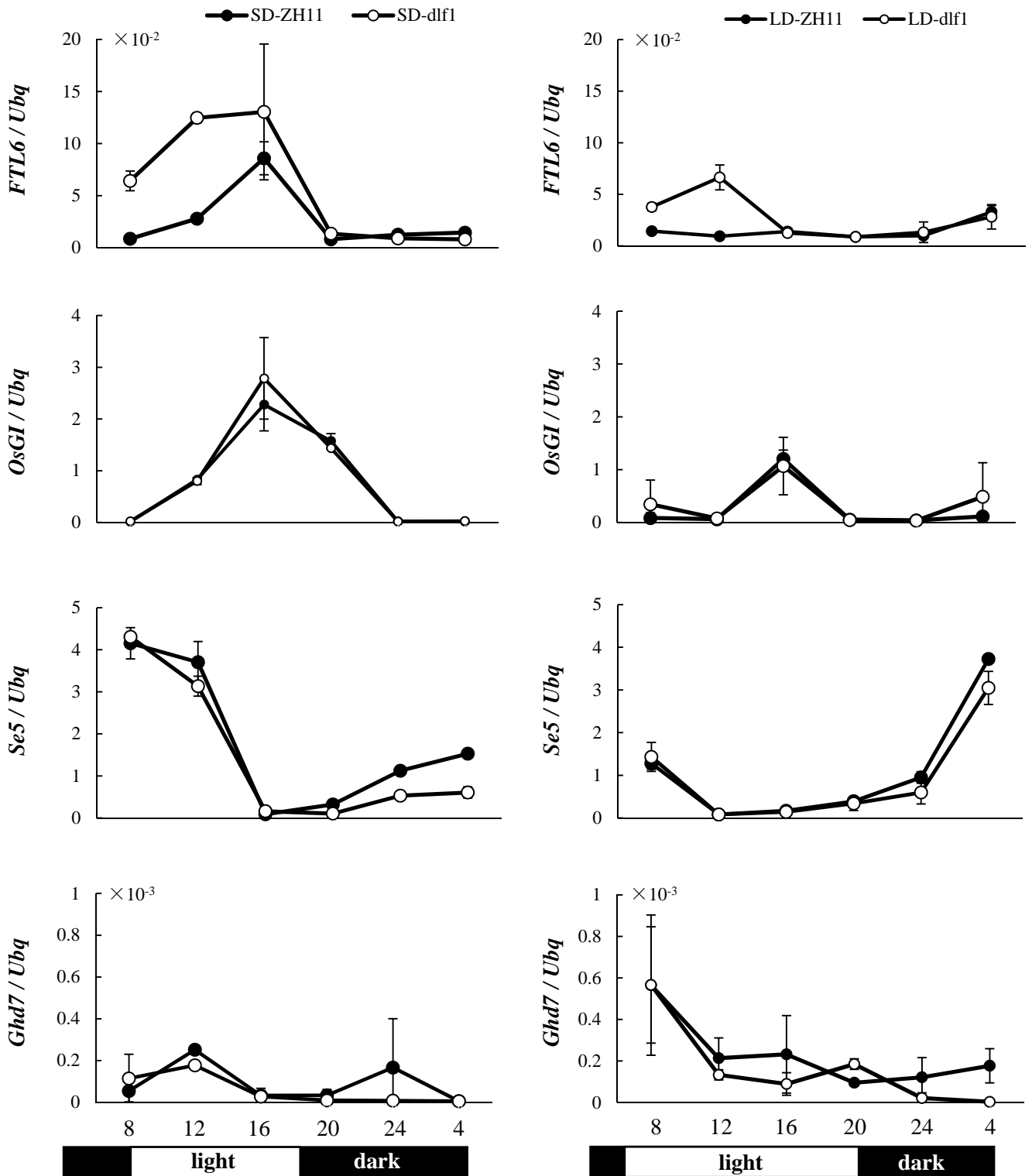

Supplemental Figure S3

Supplement: Figure S3 — Expression of OsGI , Ghd7, Se5 , and FTL6 . Diurnal expression patterns of OsGI, Ghd7, Se5, and FTL6 in the ZH11 control (filled circle) and the mutant dlf1 (open circle) plants under SD (10 h light/14 h dark) and LD (14 h light/10 h dark) conditions by qPCR analysis. The expression levels are relative to the ubiquitin (Ubq) mRNA. Values are shown as means ± SD of two independent experiments. The open and filled bars at the bottom represent the light and dark periods, respectively. (PDF) [file pone.0102529.s003.pdf]

**A**

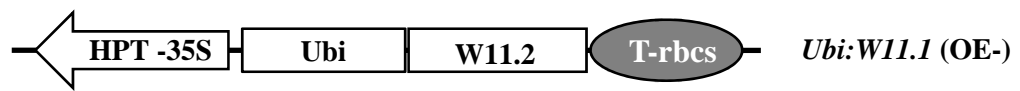

**B**

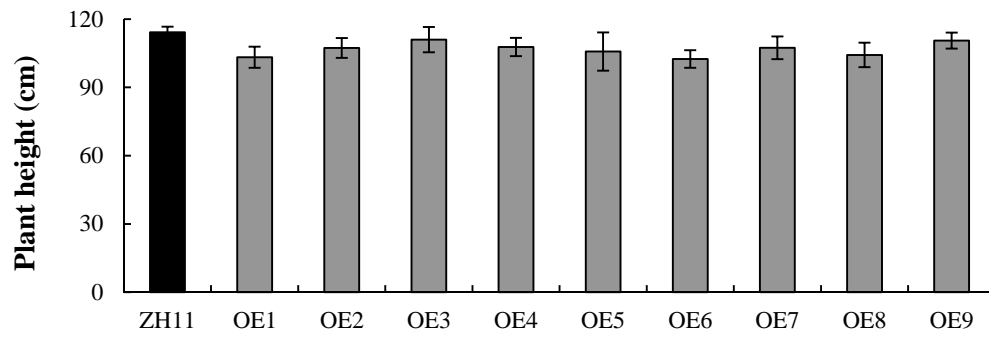

Supplement: Figure S4 — Heights of Ubi:W11.1 transgenic plants. (A) Schematic diagram of Ubi:W11.1 construct (OE-). (B) Plant heights of some Ubi:W11.1 lines in T2 progenies and the ZH11 control. Values are means ± SD. (PDF) [file pone.0102529.s004.pdf]
