# Supplementary material for: Dlf1, a WRKY Transcription Factor, Is Involved in the Control of Flowering Time and Plant Height in Rice
Source: PLoS One. 2014 Jul 18;9(7):e102529. doi: 10.1371/journal.pone.0102529 (PMC4103817; doi:10.1371/journal.pone.0102529)
Supplement: Table S1 — Primers of Dlf1 for genotype, expression, and vector construction. (DOCX) [file pone.0102529.s005.docx]

**Table S1** Primers of *Dlf1* for genotype, expression, and vector construction.

Primer name Sequence (5’ - 3’)

AD1 ngtcga(g/c)(a/t)gana(a/t)gaa

AD2 gtncga (g/c)(a/t)cana(a/t)gtt

AD3 (a/t)gtgnag(a/t)ancanaga

d40B1F taaggatcccgtgtacagcagcagcgacatg

HygF tcggcaatgagatatgaaaaagc

HygR agcttgtcgatcgacagatcc

InrBI (Forward) actggatccctcctcacttccacgcctctc

PddSalF tacgtcgacactggccggtcttatacct

PddBIR ttaggatccgggttgctgctgggcattgtg

pWU467 tatgccagcgtcaagtagtatgtt

PosddB2R ccagatcttggctgaagaagaagctctc

TR1 (Forward) ccaacagttgcgcagcctgaatg

TR2 (Forward) gctagagcagcttgagcttggat

TR3 (Forward) ttgtcgtttcccgccttcag

W1(Forward) tttccacacataagctagctcc

W4 (Reverse) atcagtcatcatcatgggttg

W5 (Reverse) ttgataaattccgggtgctagt

W6 (Reverse) cggtttcttggagttctctcca

W10SH3r tacaagcttgggtttgcggcggctcg

W10H3r tagaagcttcatcatcatgggttgctgctg

5’ RACE Outer Primer catggctacatgctgacagccta

5’ RACE Inner Primer cgcggatccacagcctactgatgatcagtcgatg

PW10 tacgttgacactggccggtcttatacc
