# Supplementary material for: Dlf1, a WRKY Transcription Factor, Is Involved in the Control of Flowering Time and Plant Height in Rice
Source: PLoS One. 2014 Jul 18;9(7):e102529. doi: 10.1371/journal.pone.0102529 (PMC4103817; doi:10.1371/journal.pone.0102529)
Supplement: Table S2 — Primers of photoperiod- and flowering-time-related genes for real-time RT-PCR. (DOCX) [file pone.0102529.s006.docx]

**Table S2** Primers of photoperiod- and flowering-time-related genes for real-time RT-PCR.

Primer name Forward (5’ - 3’) Reverse (5’ - 3’) amplified size (bp)

Ubq aaccagctgaggcccaaga acgattgattta accagtccatga 77

Hd1 tcagcaacagcatatctttctcatca tctggaatttggcatatctatcacc 80

Hd3a gctcactatcatcatccagcatg ccttgctcagctatttaattgcata 118

Ehd1 ggatgcaaggaaatcatgga aatcccatcggaaatcttgg 121

Ehd2 aacgacgacaatagctcgatcg tcacggagccttccgaggtaag 91

FTL6 tacaacagactatttatgcacc tctggcaattga aga acactgc 111

Ghd7 aggtgctacgagaagcaa atcc gggcctcatctcggcatag 108

OsGI tgcttgttgatggtgaagcttg gatagacggcacttcagcagat 145

Se5 agtcgttctcgtattctggag tctgtctgactgacgtgaact 123

Dlf1(W2/W3) agctactaccggtgcacga cccttcgtacgtggtgat 96
