# Supplementary material for: Dlf1, a WRKY Transcription Factor, Is Involved in the Control of Flowering Time and Plant Height in Rice
Source: PLoS One. 2014 Jul 18;9(7):e102529. doi: 10.1371/journal.pone.0102529 (PMC4103817; doi:10.1371/journal.pone.0102529)
Supplement: Table S3 — Primers for transactivation activity. (DOCX) [file pone.0102529.s007.docx]

**Table S3** Primers for transactivation activity.

Primer name Sequence (5’ - 3’) usage

W10EI gcgaattcatgtcttctggaggagg pBD-WRKY11.1 and the 3’ deleted

ddSal aagtcgactgggttgctgctgggcattgt pBD-WRKY11.1 and the 5’ deleted

W10-2Sal tagtcgacgggtttgcggcggctc pBD-WRKY11.2

W10N1 tagaattcgccggcggtgccgac pBD-dN1

W10N2 tagaattcgtcgccggagaaggc pBD-dN2/pBD-dC1

W10N3 gtgaattcctcgaggacggctacc pBD-dN3

W10C2 tagtcgacgctcgccgggctgtggt pBD-dC2

W10C1 tagtcgacgctcgccgggctgt pBD-dC1/pBD-dN2

.
